# The Src-Family Kinase Inhibitor PP2 Rescues Inducible Differentiation Events in Emergent Retinoic Acid-Resistant Myeloblastic Leukemia Cells

**DOI:** 10.1371/journal.pone.0058621

**Published:** 2013-03-15

**Authors:** Holly A. Jensen, Lauren E. Styskal, Ryan Tasseff, Rodica P. Bunaciu, Johanna Congleton, Jeffrey D. Varner, Andrew Yen

**Affiliations:** 1 School of Chemical and Biomolecular Engineering, Cornell University, Ithaca, New York, United States of America; 2 Department of Biological Engineering, Cornell University, Ithaca, New York, United States of America; 3 Department of Biomedical Sciences, Cornell University, Ithaca, New York, United States of America; Georg Speyer Haus, Germany

## Abstract

Retinoic acid is an embryonic morphogen and dietary factor that demonstrates chemotherapeutic efficacy in inducing maturation in leukemia cells. Using HL60 model human myeloid leukemia cells, where all-*trans* retinoic acid (RA) induces granulocytic differentiation, we developed two emergent RA-resistant HL60 cell lines which are characterized by loss of RA-inducible G1/G0 arrest, CD11b expression, inducible oxidative metabolism and p47^phox^ expression. However, RA-treated RA-resistant HL60 continue to exhibit sustained MEK/ERK activation, and one of the two sequentially emergent resistant lines retains RA-inducible CD38 expression. Other signaling events that define the wild-type (WT) response are compromised, including c-Raf phosphorylation and increased expression of c-Cbl, Vav1, and the Src-family kinases (SFKs) Lyn and Fgr. As shown previously in WT HL60 cells, we found that the SFK inhibitor PP2 significantly increases G1/G0 cell cycle arrest, CD38 and CD11b expression, c-Raf phosphorylation and expression of the aforementioned regulators in RA-resistant HL60. The resistant cells were potentially incapable of developing inducible oxidative metabolism. These results motivate the concept that RA resistance can occur in steps, wherein growth arrest and other differentiation events may be recovered in both emergent lines. Investigating the mechanistic anomalies in resistant cell lines is of therapeutic significance and helps to mechanistically understand the response to retinoic acid’s biological effects in WT HL60 cells.

## Introduction

Retinoids, the family of vitamin A derivatives, have long been known to control differentiation processes and have similar mechanisms to those of steroid and thyroid hormones [1]. Retinoic acid (RA) has pro-differentiative and anti-proliferative effects, and is associated with embryonic development, maintenance of epithelial linings and prevention of epithelial tumorigenesis [1]. RA is the current treatment for acute promyelocytic leukemia (APL) [2], and retinoids serve preventative and therapeutic roles in other cancers and diseases [3], [4]. However, RA-treated myeloid leukemia cells, and RA-treated patients, may develop RA resistance after continual treatment. Many RA-upregulated proteins may continue to be expressed in RA-resistant lines, indicating that during RA resistance certain signaling pathways remain responsive while others do not. For example, RA-dependent upregulation of the surface marker CD38 is observed in both wild-type and RA-resistant HL60 (this study) and NB4 cells [5].

The HL60 cell line is an attractive, comprehensive model for understanding how RA-induced differentiation and proliferation mechanisms operate. These myeloblastic (FAB M2) leukemia cells have been a durable experimental system since the late 1970s [6]. HL60 cells are bipotent [7] myelomonocytic precursors, capable of being induced to differentiate into monocytes or granulocytes [8], [9]. Treatment with all-*trans* retinoic acid (RA) induces differentiation of HL60 along the granulocytic lineage into neutrophil-like cells [8], [9].

In HL60, inducer treatment results in G1/G0 cell cycle arrest and the cells become committed to terminal differentiation. With a doubling time of 20–24 h, HL60 undergo two rounds of cell division after RA treatment and are committed to granulopoiesis by 48 h [10]. RA-induced HL60 cells characteristically upregulate various surface proteins, including CD38 and CD11b. CD11b is an integrin component expressed in neutrophils [11]. CD38, an extremely early marker of RA-induced differentiation [12], [13], is a nexus for many signaling proteins also upregulated with RA treatment in these cells. Intracellular binding partners of CD38 include Vav1, c-Cbl, Slp76 [14], and the Src-family kinase (SFK) Lyn [15]. Ectopic overexpression of either Vav1 [16], c-Cbl [17] or Slp76 combined with c-FMS [18] has been shown to enhance RA-induced differentiation in HL60. Also following differentiation, RA-treated HL60 cells display an inducible reactive oxygen species (ROS) response, which is a late, functional marker of mature myeloid cells [19], [20]. Another known feature correlated with myeloid differentiation in RA-induced HL60 cells is sustained activation of the Raf/MEK/ERK signaling axis, also known as the mitogen-activated protein kinase (MAPK) phosphorylation cascade [21], [22].

We recently verified in Congleton et al. (2012) [23] that the SFK inhibitor PP2 is able to enhance the RA-induced differentiation of HL60 cells. This effect was reported previously in both HL60 and NB4 cells [24] **.** PP2 is a pyrazolopyrimidine compound that is a potent inhibitor for all SFK members [25], [26]. Lyn and Fgr are the predominant kinases of this family in myeloid cells [27], [28]. Although both Lyn and Fgr are upregulated with RA treatment in HL60 cells, Lyn is the predominant SFK phosphorylated in RA-induced HL60 cells [23], [29]. This, and the existence of a PP2-induced Lyn/c-Raf interaction in HL60 cells [23], implicates Lyn as a key component of the differentiation process in these cells. Therefore, although there are potential off-target effects, PP2 treatment may enhance the induced granulocytic differentiation of HL60 leukemia cells through a Lyn-dependent process.

In this study, we first introduce and characterize two novel RA-resistant HL60 cell lines. These RA-resistant HL60 cells do not arrest in G1/G0, upregulate CD11b nor display an inducible ROS response after RA treatment. However, one RA-resistant HL60 line continues to express CD38 after RA treatment (R38+) while the sequentially emergent line has lost this ability (R38−). Both R38+ and R38− display sustained MEK/ERK activation 48 h after RA treatment, but have lost the corresponding increased c-Raf phosphorylation seen in RA-induced wild-type (WT) HL60. Both RA-resistant lines also fail to upregulate the signaling proteins Vav1, c-Cbl, Lyn and Fgr after RA treatment. We show that these signaling events can be recovered in the RA-resistant cells with PP2 treatment. PP2 also recovers aspects of RA-induced differentiation, in particular G1/G0 arrest and CD11b expression.

## Materials and Methods

### Cell Culture

**Figure 1:**
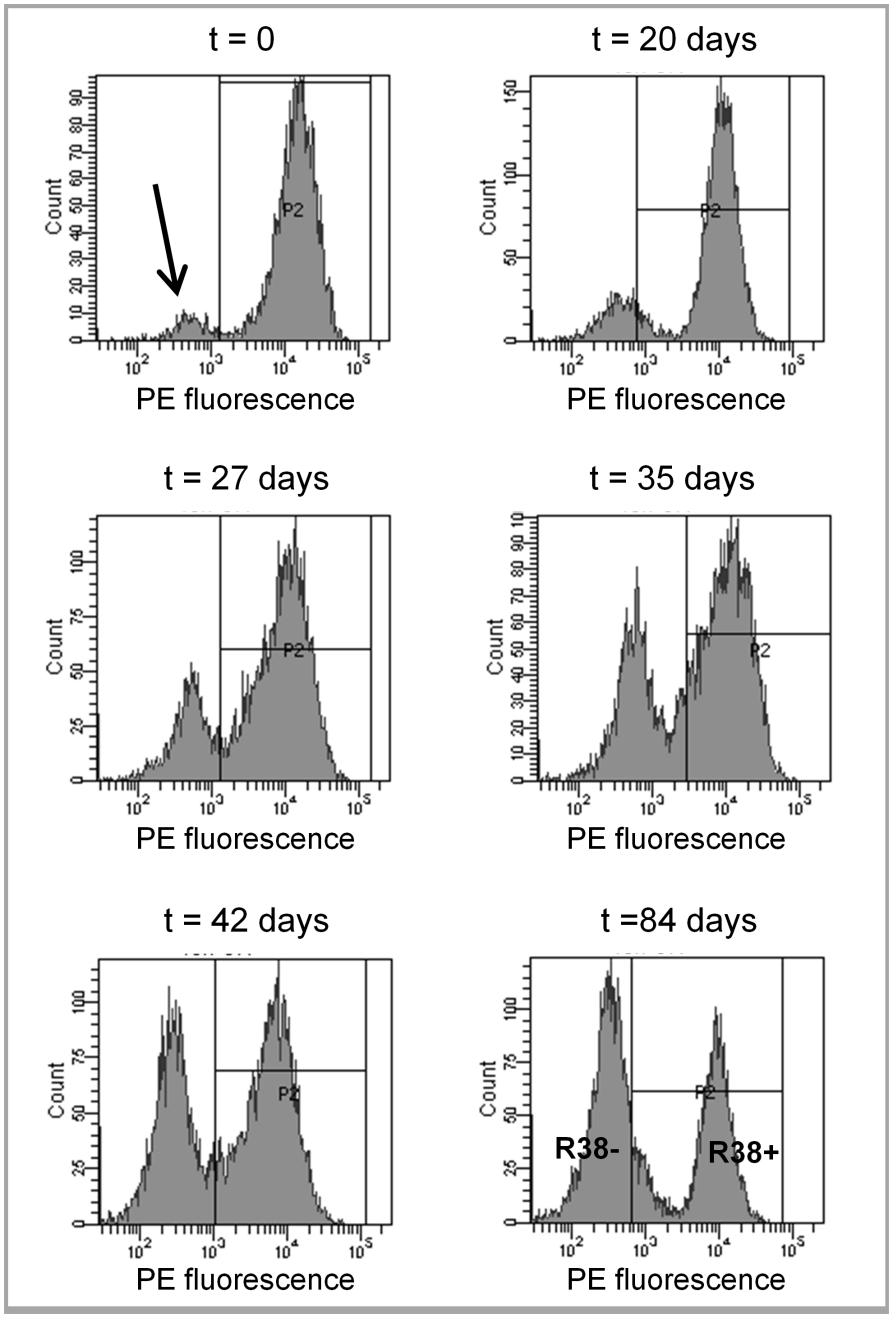
48 h PE fluorescence (CD38 surface expression) histograms for RA-resistant HL60 cells over 84 days. RA-resistant HL60 cells were labeled with PE-conjugated anti-CD38 and analyzed by flow cytometry. RA-resistant HL60 grown continuously in RA initially display elevated CD38 expression (histogram located in the gate P2). A growing subpopulation that does not express CD38, despite continual RA exposure, is indicated (by an arrow) as a low signal peak (top left graph). Over the course of 84 days, the number of cells that do not express CD38 continues to increase. The two distinct RA-resistant HL60 cells lines (bottom right graph), one with RA-inducible CD38 expression and one that has lost RA-inducible CD38 expression, were separated using Fluorescent Activated Cell Sorting (FACS) and the separated cell lines were termed R38+ and R38−, respectively.

HL60 cells were derived from the original isolates and were a generous gift of Dr. Robert Gallagher [6]; the cells were maintained in this laboratory and published by this laboratory previously [10], [12], [14], [17], [18], [21], [22], [23]. HL60 cells are cultured in RPMI 1640 medium (Invitrogen, Carlsbad, CA) supplemented with 5% FBS (Hyclone, Logan, UT) and Antibiotic-Antimycotic (Invitrogen) in a 5% CO_2_ humidified environment at 37°C. Cell passages are initiated at 0.2×10^6^ cells/ml and cell viability is monitored via Trypan Blue (Invitrogen) staining. All-*trans* retinoic acid (RA) (Sigma, St. Louis, MO) is added at 1 µM and PP2 (EMD Chemicals, San Diego, CA) is added at 10 µM. Stock RA is maintained in ethanol at 5 mM and PP2 is maintained in dimethyl sulfoxide (DMSO) at 10 mM. RA-resistant HL60 cells were established by continual growth in 1 µM RA-treated media. During continual growth in RA, cells were sorted using allophycocyanin (APC)-conjugated anti-CD38 antibody (Invitrogen) three times once a week with Fluorescence Activated Cell Sorting (BD FACSAria, BD Biosciences, San Jose, CA) to isolate CD38 positive cells that remained viable in long term RA exposure (R38+). A second RA-resistant line (R38−) emerged in time from R38+ as evidenced by a growing CD38 unshifted peak during cytometry experiments (Figure 1). This second RA-resistant line was isolated by Fluorescence Activated Cell Sorting using CD38 antibody and two-way sorting.

### Flow Cytometry

0.5×10^6^ cells were immunostained with phycoerythrin (PE)-conjugated anti-CD38 antibody and allophycocyanin (APC)-conjugated anti-CD11b antibody (BD Pharmingen, San Jose, CA) and analyzed by flow cytometry (BD LSRII flow cytometer, BD Biosciences, San Jose, CA) as described previously [17]. Control was set to exclude 95% of the live cell population peak. For cell cycle analysis, 0.5×10^6^ cells were analyzed by flow cytometry as described previously [17]. Controls were gated to approximately 45% G1/G0 phase, 35% S phase and 20% G2/M phase. The resistant cells remained diploid.

### Nitroblue Tetrazolium Reduction Assay

1×10^6^ cells were resuspended in 500 µl of 2 mg/ml nitroblue tetrazolium (NBT) (Sigma) in PBS containing either 200 ng/ml 12-O-tetradecanoylphorbol-13-acetate (TPA) (Sigma) or equivalent volume of DMSO carrier. After 1 h incubation at 37°C, samples were resuspended in 200 µl of 37% HCl (12 M). Absorbance was read at 595 nm. Stock NBT was maintained between 25 and 35 mg/ml in DMSO.

### Western Blot Analysis

Cells were washed and resuspended in M-PER lysis buffer (Thermo Scientific, Rockford, IL) supplemented with 1/100 volume of protease and phosphatase inhibitors (Sigma). Equal amounts of protein lysates (25 µg) were resolved by SDS-PAGE and transferred onto PVDF membrane (Millipore, Billerica, MA), followed by blocking (5% dry milk protein in PBS-Tween) and probed with antibodies. For immunoprecipitation, equal protein amounts (300 µg) were precleared with 30 µl protein A/G beads (Santa Cruz Biotechnology, Santa Cruz, CA), then retreated with 1/100 volume antibody and 30 µl beads overnight at 4°C before gel loading. Primary antibodies used were specific against ERK1/2, phospho(T202/Y204)-ERK1/2, MEK1/2, phospho(T217/221)-MEK1/2, Raf1, phospho(S259)-Raf1, phospho(S289/296/301)-Raf1, Lyn, Fgr, Slp76, pan-phospho(Y416)SFK, Vav1, GAPDH (Cell Signaling, Danvers, MA), c-Cbl (Santa Cruz Biotechnology), and phospho(S621)-Raf1 (Thermo Scientific). Membranes were incubated with horseradish peroxidase-linked anti-rabbit IgG secondary antibody (Cell Signaling) for 1 hour before development on film using an ECL Detection kit (Thermo Scientific). Blots shown are representative of the typical blot, repeated at least three times.

### Wright Staining

0.1×10^6^ cells were cytospun for 3 min at 700 rpm onto glass slides. Slides were then air-dried and stained with Wright’s stain. Slide images were captured at 40× (Leica DM LB 100T microscope, Leica Microsystems) using a digital camera (Olympus DP70, Olympus).

## Results

### Emergence of R38− from R38+

RA-resistant cells were established by continual growth of wild-type (WT) HL60 in RA-treated medium combined with isolation of viable cells using Fluorescence Activated Cell Sorting. The emergent RA-resistant line, R38+, retained RA-inducible expression of CD38. A second RA-resistant line (R38−) arose from the R38+ line as evidenced by a growing CD38 unshifted peak during cytometry experiments (Figure 1, see arrow in top left graph). This CD38 null RA-resistant line was also isolated by Fluorescence Activated Cell Sorting. R38+ and R38− were then removed from RA-treated media to clearly assess RA-induced signaling as compared to the WT HL60 cells.

### Characterization of Two Resistant HL60 Cell Lines

**Figure 2:**
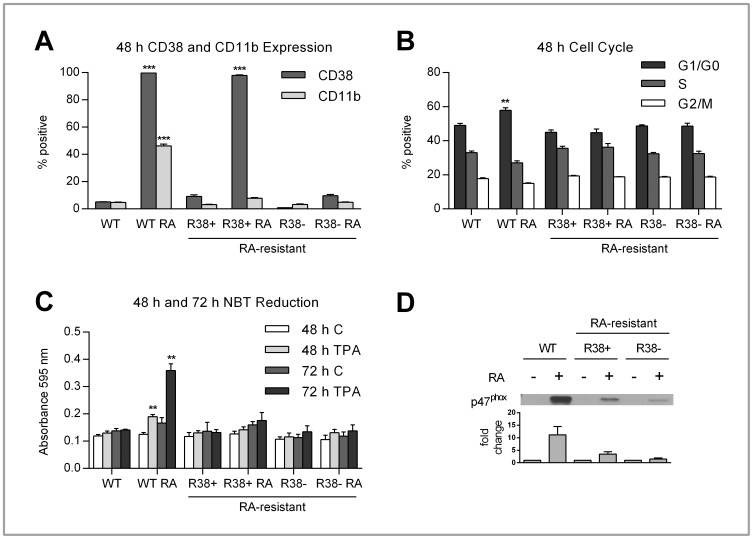
CD38 and CD11b expression, cell cycle progression, inducible ROS production and p47^phox^ expression in control and RA-treated WT, R38+ and R38− HL60 cells. Error bars represent standard error of at least three repeats. P values were calculated using a student’s t test and are compared between untreated respective control unless otherwise indicated. A: Control and RA-treated WT, R38+ and R38− HL60 cells were labeled with PE-conjugated CD38 and APC-conjugated CD11b and analyzed by flow cytometry. To measure the percent positive signal, WT control was set to exclude 95% of the live cell population peak. RA-treated WT HL60 displayed significant (p<<0.001) increased CD38 (∼98%) and CD11b (∼45%) protein surface expression after 48 h. RA-treated R38+ also significantly (p<<0.001) upregulated CD38 (∼98%) surface expression at 48 h. However, neither RA-treated R38+ nor R38− displayed any significant upregulation of CD11b at 48 h. B: To assess cell cycle progression, control and RA-treated WT, R38+ and R38− HL60 cells were stained with propidium iodide-hypotonic solution and analyzed by flow cytometry. Controls were gated to approximately 45% G1/G0 phase, 35% S phase and 20% G2/M phase. RA-treated WT HL60 cells exhibited significant (p<0.01) increased G1/G0 arrest (∼60%) after 48 h. However, neither RA-treated R38+ nor R38− exhibited any increased G1/G0 growth arrest at 48 h. C: To assess ROS production, control and RA-treated WT, R38+ and R38− HL60 cells were stimulated with TPA and analyzed by NBT reduction. At 48 h and 72 h, only RA-treated WT HL60 cells displayed a significant ROS response (p<0.01 compared to both control and RA-treated resistant cells) after TPA induction. Neither RA-treated R38+ nor R38− was able to exhibit ROS production after stimulation with TPA at either 48 or 72 h. D: RA-treated WT HL60 cells showed enhanced p47^phox^ expression after 48 h, whereas only minimal p47^phox^ expression is induced by RA in R38+ and R38−.

In WT HL60 cells, RA treatment results in increased CD38 (∼98%) and CD11b (∼45%) surface expression at 48 h (Figure 2A). The first RA-resistant HL60 cell line, termed R38+, also shows ∼98% increase in CD38 expression after 48 h of RA treatment. The second RA-resistant line, R38−, shows no RA-induced CD38 expression after 48 h (Figure 2A). Neither of the RA-resistant HL60 cell lines were able to upregulate CD11b, a slightly later differentiation marker, 48 h after RA treatment (Figure 2A). The percentage of cell population in the G1/G0, S, and G2/M phases of cell cycle was investigated. It is known that for RA-treated WT HL60, onset of G1/G0 arrest occurs after 48 h [21], [23]. Only WT HL60 cells showed any significant increase in the percentage of cell population arrested in the G1/G0 phase of cell cycle 48 h after RA treatment (Figure 2B). At the later timepoints 72 h and 96 h (not shown) only the WT HL60 cells continue to exhibit growth arrest after RA treatment.

The ability to induce reactive oxygen species (ROS) production was determined by stimulation with 12-O-tetradecanoylphorbol-13-acetate (TPA). Reduction of nitroblue tetrazolium (NBT) into a blue formazan product in the presence free radical oxygen served as the reporter of ROS production. At 48 h, only RA-treated WT HL60 cells display an inducible ROS production response (Figure 2C). We also assessed NBT reduction at 72 h, and this confirmed that by 72 h, RA-treated WT HL60 cells are capable of inducible ROS production, whereas the RA-resistant HL60 cells have lost this function. In addition to NBT reduction, the expression of the p47^phox^ protein was examined. The p47^phox^ protein is a component of the NADPH oxidase complex, which is responsible for generating free oxygen radicals [20]. The upregulated expression of p47^phox^ after RA-treatment is greatly diminished in both R38+ and R38− (Figure 2D). Meanwhile, RA-treated WT HL60 cells show striking upregulation of p47^phox^ at 48 h. In conclusion, the lack of CD11b expression, G1/G0 cell cycle arrest and inducible ROS production verifies that both R38+ and R38− HL60 cell lines are indeed resistant to treatment with RA.

### Expression and Activation of Intracellular Signaling Proteins

**Figure 3:**
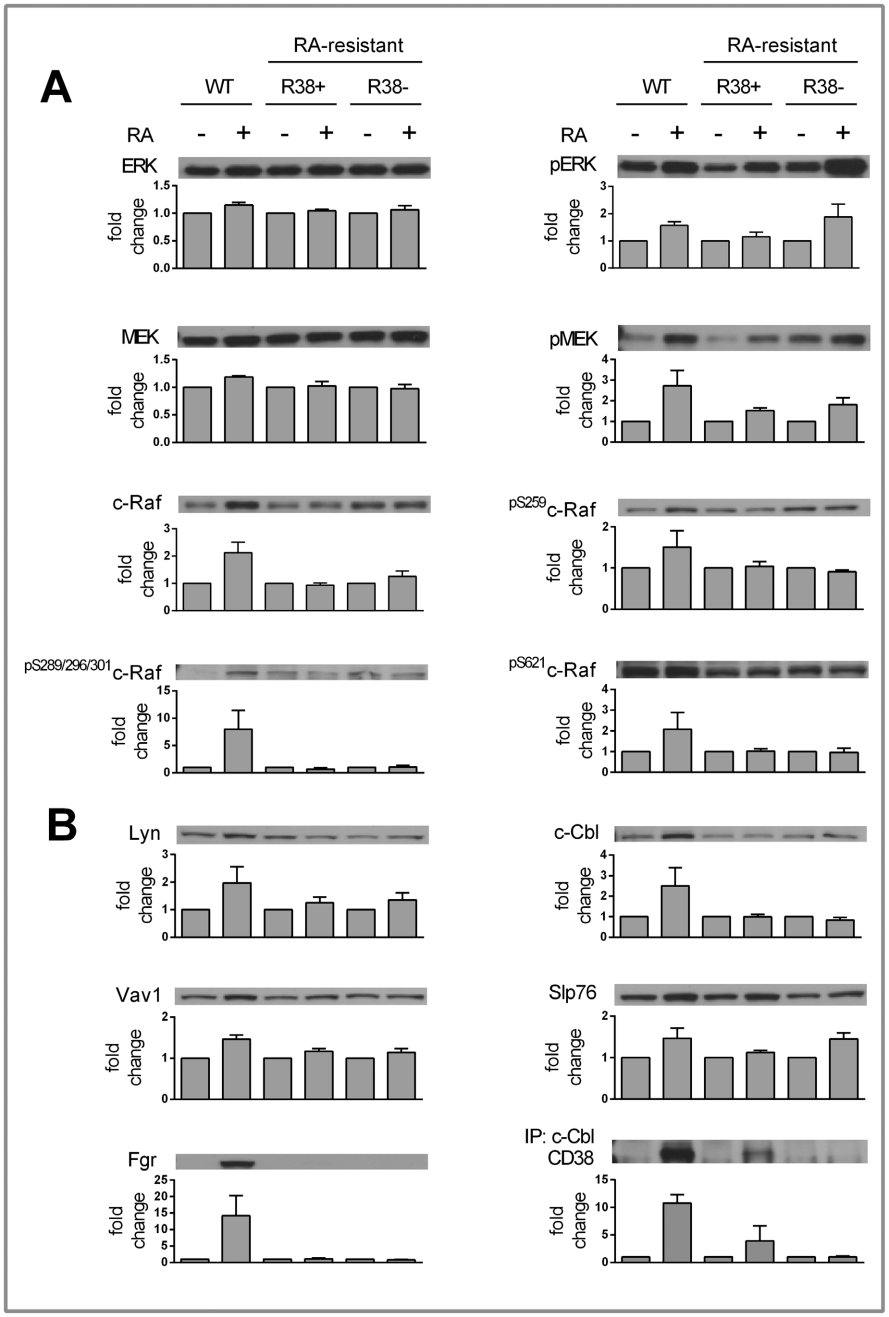
48 h Western blot data for control and RA-treated WT, R38+ and R38− HL60 cells. A representative blot is displayed above its respective bar graph, and each bar graph (error bars represent standard error) presents the fold change respective to each control. The fold change was calculated after performing densitometry across three or more repeated blots. Note that the scale of the y-axis for each bar graph differs. A: There was no change in total ERK or MEK levels for any cell line. RA induced MEK and ERK phosphorylation in all three cell lines. Only RA-treated WT HL60 cells showed upregulation of c-Raf expression. Also, only RA-treated WT HL60 cells exhibited increased c-Raf phosphorylation at S259, S621 and S289/296/301. Neither R38+ nor R38− displayed increased c-Raf expression or phosphorylation after RA treatment. B: RA-treated WT HL60 cells showed upregulation of Lyn, Fgr, Vav1, and c-Cbl expression. RA-inducible Slp76 expression was evident in R38+ and R38−. Immunoprecipitation of c-Cbl followed by blotting of CD38 reveals that there is little CD38 and c-Cbl interaction in RA-treated R38+ compared to RA-induced WT HL60. GAPDH (not shown) served as loading control; c-Cbl (not shown) served as control for c-Cbl immunoprecipitation.

Sustained activation of the Raf/MEK/ERK proteins is a long-known feature of RA-treated HL60, and direct inhibition of MEK (and subsequently ERK as well as c-Raf) using PD98095 has been shown to abolish RA-induced differentiation of HL60 [21], [22]. We assessed whether sustained activation of MEK and ERK occurs with RA treatment in RA-resistant HL60 cells, and found that indeed MEK and ERK always show increased phosphorylation 48 h after RA treatment in both R38+ and R38− cells (Figure 3A). Total levels of MEK and ERK remained unchanged after RA treatment.

We next investigated c-Raf expression, which increases with RA treatment in WT HL60, but this increase is absent in the RA-resistant cells (Figure 3A). We also determined the phosphorylation of the S259, S621, and collectively the S289/296/301 sites on c-Raf after 48 h of RA treatment. Consistent with c-Raf expression, phosphorylation at these sites on c-Raf is increased 48 h after RA treatment in WT HL60 cells, but does not increase in R38+ or R38− (Figure 3A), despite concurrent MEK/ERK activation. It appears that phosphorylation at these c-Raf sites is uncoupled from MEK/ERK activation in the RA-resistant cells (see Discussion).

Signaling proteins known to interact with CD38 are upregulated during RA-induced differentiation in WT HL60 cells; these include c-Cbl, Vav1, Slp76 [14] and Lyn [15]. In RA-resistant HL60 cells, Slp76 was the only one of these factors that might be upregulated 48 h after RA treatment (Figure 3B). For Vav1, c-Cbl and Lyn, RA-induced expression was prominent only for the WT HL60 across all repeats. Also, we found that Fgr is similarly upregulated in RA-induced WT HL60 but does not increase in the resistant cells. An immunoprecipitation of c-Cbl reveals that in RA-treated R38+ (which express CD38 after RA treatment), there was reduced c-Cbl interaction with CD38 compared to RA-treated WT HL60 (Figure 3B).

### PP2 Rescues the Differentiation of RA-resistant HL60

**Figure 4:**
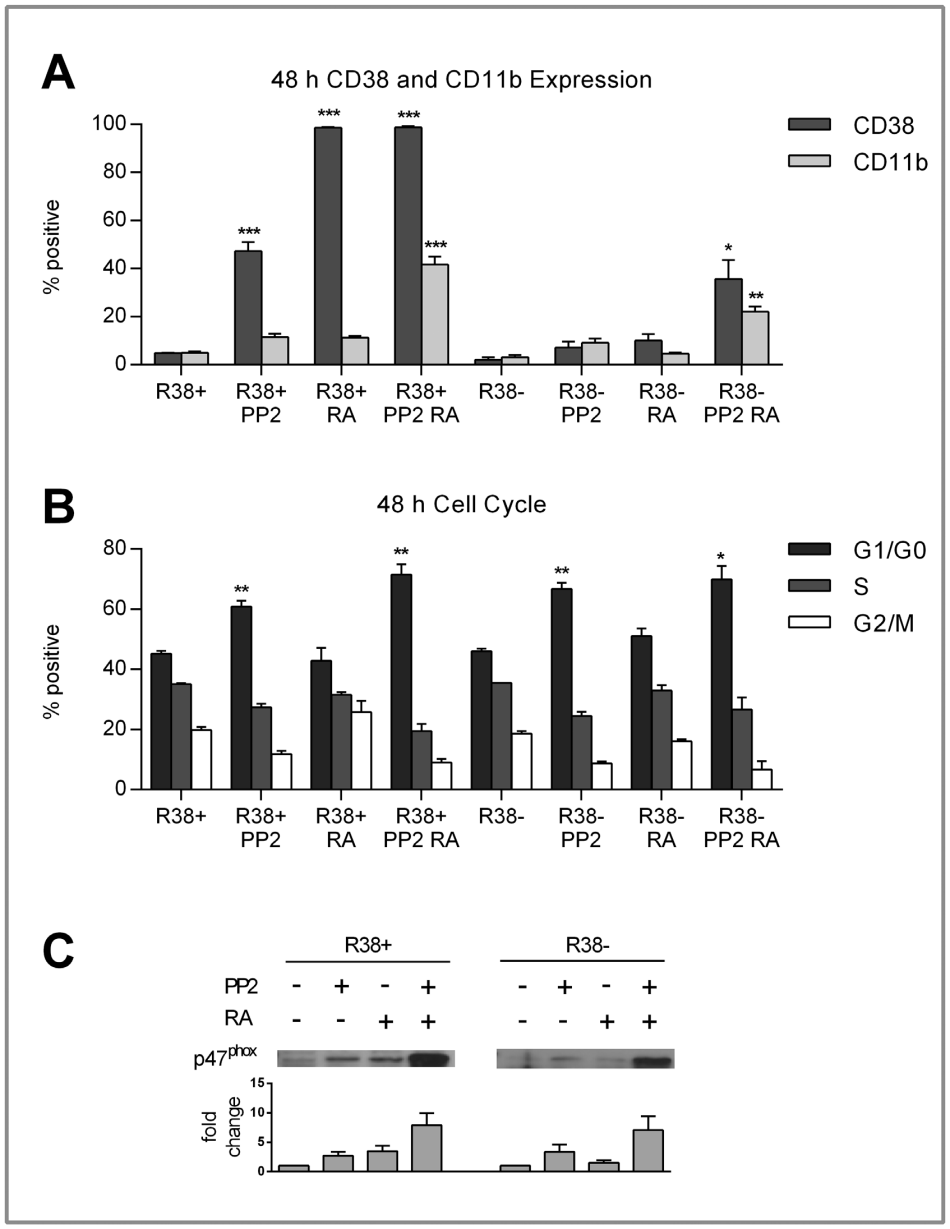
CD38 and CD11b expression, cell cycle progression, and p47^phox^ expression in control, PP2- and/or RA-treated R38+ and R38− HL60 cells. Error bars represent standard error of at least three repeats. P values were calculated using a student’s t test and are compared between untreated respective control unless otherwise indicated. A: Control, RA-treated and/or PP2-treated R38+ and R38− HL60 cells were labeled with PE-conjugated CD38 and APC-conjugated CD11b and analyzed by flow cytometry. To measure the percent positive signal, controls were set to exclude 95% of the live cell population peak. After 48 h, PP2 induced significant (p<0.001) CD38 expression (∼50%) in R38+. Combined PP2+RA treatment in R38+ significantly increased (p<0.001) CD11b to levels comparable with those of RA-treated WT HL60 cells (∼40%). In R38−, co-treatment resulted in diminished yet still significant increases CD38 (p<0.05, nearly 40%) and CD11b (p<0.01, more than 20%) expression. PP2 treatment alone had no significant effect on CD38 or CD11b expression in R38−. B: To assess cell cycle progression, control and RA-treated WT, R38+ and R38− HL60 cells were stained with propidium iodide-hypotonic solution and analyzed by flow cytometry. Controls were gated to approximately 45% G1/G0 phase, 35% S phase and 20% G2/M phase. PP2 induced growth arrest (more than 60%) in both R38+ (p<0.01 alone and with RA) and R38− (p<0.01 with PP2 alone, p<0.05 with co-treatment). C: p47^phox^ expression was upregulated in both R38+ and R38− with combined PP2+RA treatment. PP2 alone did not increase p47^phox^ expression greater than RA treatment alone in either R38+ or R38−.

It was confirmed by Congleton et al. (2012) [23] that PP2 treatment alone and with RA is able to enhance differentiation markers (CD11b, G1/G0 arrest, p47^phox^ expression and c-Raf phosphorylation) in WT HL60 cells. This led us to ask whether PP2 could rescue similar events in the RA-resistant HL60 cells. We found that, in the R38+ cells, combined PP2+RA treatment could increase CD11b expression to levels comparable with RA-induced WT HL60 cells (∼40%) after 48 h (Figure 4A). Since CD38 is already maximally expressed with RA treatment in R38+ cells, combined PP2+RA treatment could not enhance CD38 expression further. However, PP2 alone could induce some (∼50%) CD38 expression (Figure 4A). In the R38− cells, PP2 treatment had a diminished yet visible effect on CD38 and CD11b expression, with combined PP2+RA treatment resulting in minor CD38 (∼40%) and CD11b (∼20%) increase in expression after 48 h (Figure 4A). In both RA-resistant HL60 cell lines, PP2 treatment alone and co-treated with RA enhanced the G1/G0 cell cycle arrest after 48 h (Figure 4B) to ∼60–70%.

PP2 in combination with RA can upregulate the p47^phox^ protein in both R38+ and R38− (Figure 4C). We found that, despite upregulated p47^phox^ expression seen in PP2-treated WT HL60 cells [23], PP2 treatment diminished the inducible ROS response seen in RA-treated WT HL60 (Figure S1B). As expected, PP2 was potentially incapable of rescuing the inducible ROS production in both R38+ and R38− cell lines (Figure S1A). However, this may not be indicative of incomplete functional differentiation (see Discussion).

### Expression and Activation of Intracellular Signaling Proteins with PP2

**Figure 5:**
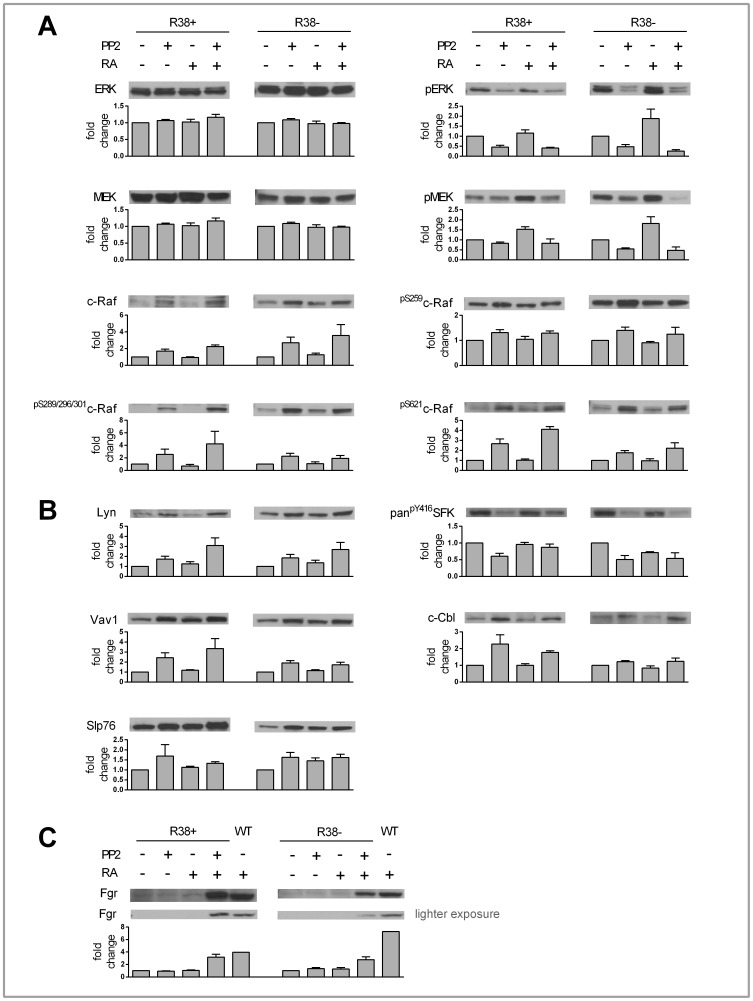
48 h Western blot data for control, PP2, RA and PP2+RA treated R38+ and R38 −**.** A representative blot is displayed above its respective bar graph, and each bar graph (error bars represent standard error) presents the fold change respective to each control. The fold change was calculated after performing densitometry across three or more repeated blots. Note that the scale of the y-axis for each bar graph differs. A: There was no change in total ERK or MEK levels during any treatment for either R38+ or R38−. Interestingly, PP2 (alone and when co-treated with RA) decreased MEK and ERK phosphorylation in both R38+ and R38−. However PP2 and PP2+RA treatment induced upregulation of c-Raf expression and c-Raf phosphorylation at S259, S621 and S289/296/301 in both R38+ and R38−. B: PP2 and PP2+RA induced upregulation of Lyn, Vav1, c-Cbl and Slp76 expression. Y416 phosphorylation was reduced with PP2 treatment. C: Fgr can be detected with combined RA and PP2 treatment, but not with PP2 alone in both R38+ and R38−. Although the blots of Fgr were repeated three or more times, the blot that additionally displays the band for RA-treated WT HL60 (in the blot shown) was performed once. GAPDH (not shown) served as loading control.

We investigated the Raf/MEK/ERK cascade and expression of CD38 binding partners after 48 h of PP2 and PP2+RA treatment in both RA-resistant HL60 cell lines. Intriguingly, the presence of PP2 grossly cripples the phosphorylation of ERK and MEK in the RA-resistant cells, with no change in total MEK or ERK levels (Figure 5A). In contrast to this, PP2 treatment both alone and with RA rescues c-Raf expression and c-Raf phosphorylation at S259, S621 and S289/296/301 in R38+ and R38−. Again, there is an apparent disconnect between phosphorylation at these c-Raf sites and downstream MEK/ERK phosphorylation in both RA-resistant cell lines (see Discussion). PP2 also upregulates the expression of Vav1, c-Cbl, Slp76, and Lyn (Figure 5B) in R38+ and R38−. In WT HL60, PP2 inhibits Src-family kinase (SFK) Y416 phosphorylation (LynY397), while co-treatment with RA protects phosphorylation at this site in the presence of PP2. Similar to the WT HL60, as reported by Congleton et al. (2012), Lyn phosphorylation evaluated with antibody for pan^pY416^SFK (LynY397) was decreased with PP2 treatment, but unlike WT HL60, co-treatment with both PP2+RA did not protect this phosphorylation in R38+ or R38−. Interestingly, in R38+ combined PP2+RA treatment was able to upregulate Fgr expression to a similar level as in RA-treated WT HL60 (Figure 5C). Combined PP2+RA treatment also increased Fgr expression in R38−, but to a lesser degree as compared to RA-treated WT HL60 (Figure 5C).

### Visualization of Cell Morphology

**Figure 6:**
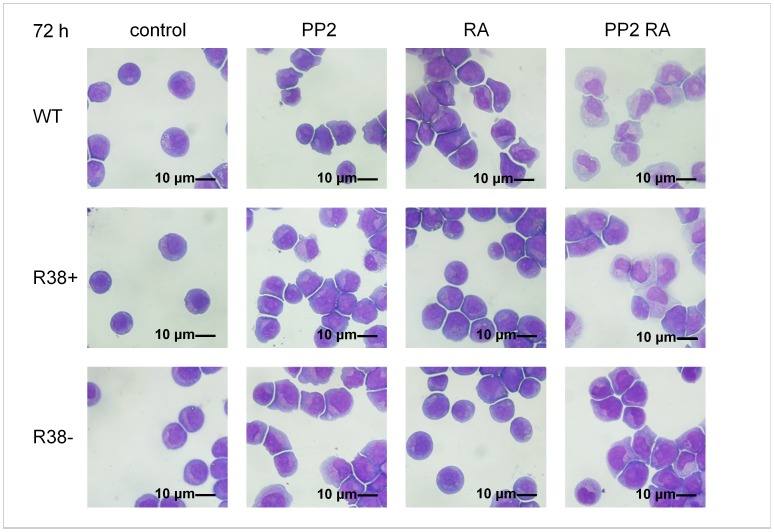
Wright’s stain cytology for control and treated WT, R38+ and R38− HL60 cell lines. Control WT cells are round; RA-treated R38+ and R38− also retain a round, stem-like appearance. WT HL60 cells showed morphological changes consistent with differentiation toward granulocytes when treated with RA, PP2, or PP2+RA. Meanwhile the two RA-resistant HL60 cell lines, R38+ and R38−, both showed morphological changes consistent with differentiation only during PP2 and PP2+RA treatment, but not RA treatment alone.

We were interested in visualizing the morphological changes that occur in RA-resistant versus WT HL60 cells after RA, PP2 or combined treatment. At 72 h, untreated control cells are round, stem-like cells with large, round to oval nuclei (Figure 6). RA-treated RA-resistant cells share the same morphology with untreated control cells. PP2 treatment in both WT and the two RA-resistant lines induced a shift from the round morphology into irregularly shaped cells with more indented nuclei characteristic of early neutrophilic differentiation. RA induction in the WT HL60 also had this effect. Combined PP2+RA treatment in WT, R38+ and R38− accelerated the morphological changes induced by PP2 alone, revealing later-stage neutrophilic differentiation.

## Discussion

### Summary of RA-resistance

The HL60 myeloblastic leukemia cell line provides a durable system for studying retinoic acid (RA)-induced differentiation mechanisms. In these cells, RA-induced terminal differentiation along the granulocytic lineage is accompanied by various phenotypic and functional changes. These include surface expression of CD38 and CD11b, growth arrest in the G1/G0 cell cycle phase, the ability to produce reactive oxygen species (ROS), upregulation of p47^phox^ and a sustained MAPK activation signal. RA treatment also induces upregulation of CD38-binding partners, including Vav1, c-Cbl, Slp76, and also the Src-family kinases (SFKs) Lyn and Fgr.

We established two RA-resistant HL60 cells lines: R38+ and R38−. RA resistance has previously been found to be attributable to mutation of the RARα gene [30], [31], [32]. However, expression of unmutated RARα did not rescue RA responsiveness in one case [30]. Meanwhile, RA resistance in various *in vitro* cell lines is not always accompanied by complete loss of internal signaling. It was found that RA-dependent upregulation of the surface marker CD38 is observed in both wild-type (WT) and RA-resistant HL60 (this study) and NB4 cells [5]. CD38, an extremely early marker of granulocytic/monocytic differentiation, contains a strong retinoic acid response element (RARE) in its first intron, to which ligand-bound retinoic acid receptor (RAR) heterodimerized with retinoid receptor (RXR) can bind and elicit transcription [3,4,l2,13]. The fact that the RAR/RXR is seemingly still functionally capable of eliciting transcription (CD38 expression) in RA-resistant HL60 cells indicates that other mechanisms emerge to confer resistance, although their nature is yet to be fully elucidated.

**Figure 7:**
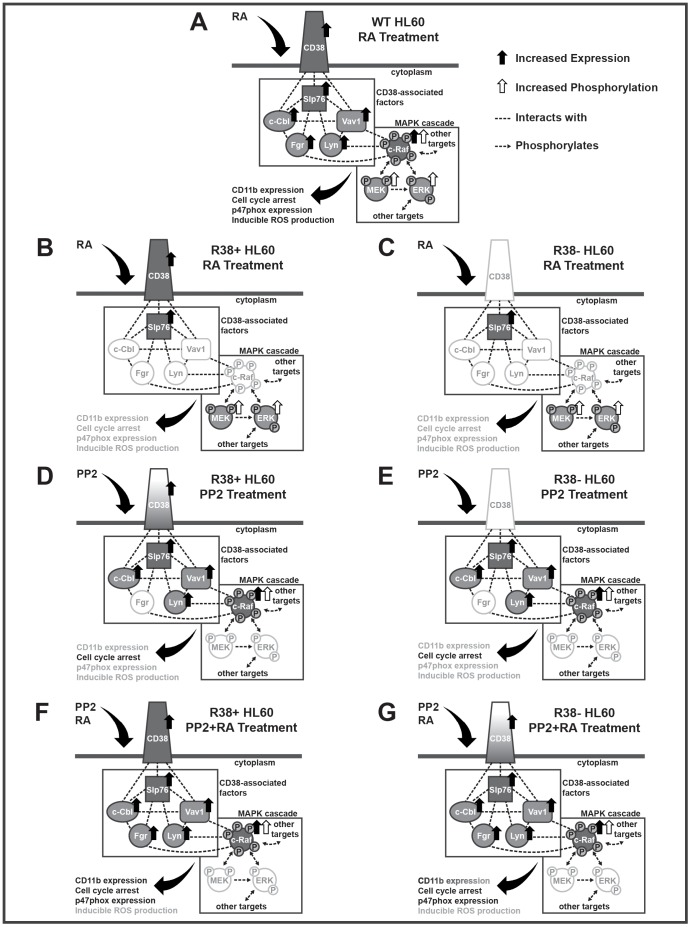
Diagrams of the signaling proteins investigated in this study for each treatment case in R38+ and R38− compared to the RA-treated WT HL60 cells. For a given protein, solid black arrows indicate increased expression while white arrows indicate increased phosphorylation. Dashed black lines indicate an interaction that has previously been demonstrated in this lab by immunoprecipitation and/or FRET, with the exception of Fgr:Slp76 and Fgr:c-Raf. These two interactions are implied by the known binding of Slp76 and c-Raf to Src-family kinase members. Dashed black lines with arrowheads indicate phosphorylation (kinase) events known in the literature. Solid-filled factors vs. white-filled factors serve to clarify the expression indicated by the black arrows. Gradient-filled factors indicate expression but to a lower level than the RA-treated WT case. Downstream effects (CD11b expression, p47^phox^ expression, etc) are written in black if they occur, grey if they do not occur, or in gradient if they occur but to a lesser extent than the RA-treated WT HL60 case. A: In RA-treated WT HL60, CD38 is upregulated, along with its intracellular binding partners Slp76, Vav1, c-Cbl, and Lyn. Fgr is also upregulated. MEK and ERK show increased phosphorylation, while c-Raf is upregulated and shows increased phosphorylation at S259, S621 and S289/296/301. Differentiation markers that occur include CD11b expression, cell cycle arrest, p47^phox^ expression and inducible ROS production. B: In RA-treated R38+ HL60, CD38 is upregulated, but not Vav1, c-Cbl, or Lyn. Fgr is not upregulated. MEK and ERK show increased phosphorylation; however c-Raf is not upregulated, nor shows increased phosphorylation. Increased CD11b expression, cell cycle arrest, p47^phox^ expression and inducible ROS production do not occur. C: In RA-treated R38− HL60, CD38 is not upregulated, nor Vav1, c-Cbl, or Lyn. Fgr is not upregulated. MEK and ERK show increased phosphorylation; however c-Raf is not upregulated, nor shows increased phosphorylation. Increased CD11b expression, cell cycle arrest, p47^phox^ expression and inducible ROS production do not occur. D: In PP2-treated R38+ HL60, CD38 is partially upregulated (indicated by the gradient CD38), and Slp76, Vav1, c-Cbl, and Lyn are upregulated. Fgr is not upregulated. MEK and ERK phosphorylation is decreased; however c-Raf is upregulated and shows increased phosphorylation. Increased cell cycle arrest occurs, but not increased CD11b expression, p47^phox^ expression or inducible ROS production. E: In PP2-treated R38− HL60, CD38 is not upregulated, but Slp76, Vav1, c-Cbl, and Lyn are upregulated. Fgr is not upregulated. MEK and ERK phosphorylation is decreased; however c-Raf is upregulated and shows increased phosphorylation. Increased cell cycle arrest occurs, but not increased CD11b expression, p47^phox^ expression or inducible ROS production. F: In PP2+RA-treated R38+ HL60, CD38 is upregulated, along with Slp76, Vav1, c-Cbl, and Lyn. Fgr is also upregulated. MEK and ERK phosphorylation is decreased; however c-Raf is upregulated and shows increased phosphorylation. Differentiation markers that occur include CD11b expression, cell cycle arrest, and p47^phox^ expression, but not inducible ROS production. G: In PP2+RA-treated R38− HL60, CD38 is partially upregulated, along with Slp76, Vav1, c-Cbl, and Lyn. Fgr is also upregulated. MEK and ERK phosphorylation is decreased; however c-Raf is upregulated and shows increased phosphorylation. Differentiation markers that occur include partial CD11b expression, cell cycle arrest, and p47^phox^ expression, but not inducible ROS production.

The response these two resistant lines exhibit to RA and/or PP2 treatment compared to RA-treated WT HL60 are diagramed in Figure 7 for clarity. Both resistant cell lines fail to respond to RA treatment in that they do not upregulate CD11b, arrest in G1/G0 nor gain an inducible ROS function or significantly upregulate p47^phox^ (Figure 7B and 7C). The R38+ cell line, however, retains RA-inducible CD38 expression while R38− has lost this ability. Both R38+ and R38− exhibit sustained ERK and MEK phosphorylation 48 h after RA treatment, but show no increase in c-Raf expression or phosphorylation as compared to RA-induced WT HL60 cells. We checked specifically for c-Raf sites that we have previously shown to be phosphorylated in RA-treated WT HL60 cells, which include the S259, S621 and S289/296/301 sites. Phosphorylation at canonical c-Raf activating sites such as S338 and Y340/341 [33], [34] cannot be detected in RA-induced WT HL60 cells [35].

The S259 site is putatively an inhibitory site [36] that prevents relocation of c-Raf to the plasma membrane [37], [38] and thus prevents its participation in membrane-initiated signaling events. The constitutively phosphorylated S621 site appears to be a stability site that maintains the kinase activity of c-Raf [36] and prevents c-Raf degradation [39]. The S289/296/301 sites are retrophosphorylated by ERK; whether this phosphorylation is inhibitory [40] or activating [41] is subject to debate. In both R38+ and R38−, RA-induced MEK/ERK activation occurs without increased c-Raf expression or phosphorylation. Therefore phosphorylation at these c-Raf sites is uncoupled from MEK/ERK activation in the RA-resistant cells. This suggests that RA may initiate MEK and ERK phosphorylation through a c-Raf-independent mechanism. It is known that retinoids can directly bind kinases like PKC and even c-Raf [42], suggesting that RA can affect signaling independent of its transcriptional effects. In NIH3T3 cells, ERK phosphorylation was found to be more extensive during c-Raf knockdown than control [43].

The RA-resistant lines also fail to exhibit increased expression of Lyn, Fgr, Vav1, or c-Cbl 48 h after RA treatment, while retaining RA-inducible Slp76 expression. Lyn and Fgr are the predominant SFKs in myeloid leukemia cells [27], [28] and Lyn binds to CD38 [15]. Vav1, expressed solely in hematopoietic cells, is upregulated in RA-induced HL60 cells [44] and serves a cytoplasmic role as an adaptor and guanine nucleotide exchange factor (GEF), as well as a nuclear role as a transcription factor and cytoskeletal remodeling protein [44], [45]. Slp76 has several protein binding domains and appears to act as an adaptor that exists in an RA-inducible CD38-assocaited complex containing Slp76/Vav1/c-Cbl [14]. Transfection of mutant G306E c-Cbl, which cannot interact with CD38, into HL60 cells eradicates RA-induced differentiation and MAPK signaling [14]. The loss of RA-induced expression of all these factors suggests that there is a wide disruption in the RA-resistant cells of signaling molecules attributed with regulatory roles in the MAPK signaling and other pathways needed to drive differentiation (Figure 7B and 7C). We speculate that there is a seminal signaling regulator of unknown identity that is disrupted in the resistant cells.

### PP2 Rescues Differentiation Markers in RA-resistant HL60 Cells

In the RA-resistant HL60 cells, PP2 did not rescue the inducible ROS production response measured by NBT reduction. However, this may not be indicative of incomplete functional differentiation. This may reflect the dependence of the neutrophil NAPDH-dependent inducible oxidative metabolism response on kinases targeted by PP2, in which case the inducible ROS results may not bear full fidelity to the degree of differentiation in the presence of this drug. In RA-treated WT HL60 cells, co-treatment with PP2 diminishes the inducible ROS response measured by NBT reduction (Figure S1B), indicating that PP2 may be inhibiting the ROS production pathway. Therefore it is unclear whether the ability to produce ROS is restored in the RA-resistant cells; upregulation of p47^phox^ during co-treatment argues that the production machinery may be intact.

Nonetheless, PP2 is able to significantly rescue G1/G0 cell cycle arrest and CD38 and CD11b surface expression in RA-resistant HL60 cells, although the rescue of CD38 and CD11b in the R38− line is diminished compared to R38+. Although CD38 expression did correlate with enhanced CD11b expression upon rescue, overall, retention of RA-inducible CD38 expression did not predict a better rescue of the resistant cells as both R38+ and R38− show a similarly positive response (G1/G0 arrest, expression of signaling factors) to PP2 treatment. PP2 thus has a similar effect on both the RA-resistant and the WT HL60 cells, in that it cannot increase the inducible ROS production response, but can promote other differentiation markers (Figure 7D–G). PP2 has previously been shown to reduce proliferation in myeloid blasts [46], and other cell types [43], [47].

PP2 was reported to enhance Ras-independent Raf phosphorylation [48]. This is consistent with our data, as we see an increase in S259 phosphorylation, which inhibits canonical Ras-induced (membrane-initiated) c-Raf activation [37]. How PP2 contributes to enhanced S259, S621 or S289/296/301 c-Raf phosphorylation is not yet understood, although we reported in Congleton et al. (2012) that RA and/or PP2 treatment induces interaction between Lyn and ^pS259^c-Raf in WT HL60. We have yet to investigate any Lyn/c-Raf interaction in PP2-treated RA-resistant cells. However, the existence of a PP2-induced Lyn/c-Raf interaction may be seminal to changes in c-Raf phosphorylation at S259 as well as the S621 and S289/296/301 sites.

Interestingly, PP2 treatment in both R38+ and R38− results in a decrease of ERK phosphorylation, an effect that has been documented in other cell lines [49], [50], [51]. This is disparate of the WT HL60 data reported [23], where PP2 treatment had no effect on MEK or ERK activation. This points to an uncoupling between c-Raf phosphorylation and MEK/ERK phosphorylation in the RA-resistant cells (Figure 7D–G). Uncoupling of c-Raf phosphorylation from downstream ERK activation during PP2 treatment has been shown previously [43].

It is unknown how PP2 enhances c-Raf phosphorylation while simultaneously decreasing MEK and ERK phosphorylation in R38+ or R38−. However, PP2-induced c-Raf phosphorylation, despite a decrease in MEK and ERK phosphorylation, is consistent in that c-Raf has defined ERK-independent functions [34], [52]. c-Raf plays a role in apoptosis that is independent of its catalytic activity [34], [53], [54] and c-Raf may serve scaffolding functions [52]. Therefore PP2 may promote MEK/ERK-independent activities of c-Raf through its effects on Lyn, which is capable of interacting with c-Raf. The reason for differences in MEK/ERK activation after PP2 treatment in WT HL60 (no effect) and the RA-resistant HL60 (decrease) is less clear.

Both PP2 and PP2+RA treatment in R38+ and R38− decreases Y416 (Lyn397) phosphorylation. However, we note that in RA-treated WT HL60 cells, Lyn phosphorylation is preserved with combined PP2+RA treatment [23]. Therefore in the PP2-treated RA-resistant cells, there is a failure for RA to protect continued phosphorylation of Lyn. Interestingly, combined PP2+RA treatment is correlated with higher CD38 and CD11b surface expression and G1/G0 cell cycle arrest in both WT HL60 (where Lyn phosphorylation is preserved) and RA-resistant HL60 (where Lyn phosphorylation is lost). This may indicate that Lyn serves a function during differentiation not dependent on this phosphorylation site. Since PP2+RA co-treatment in R38+ and R38− enhances CD38 and CD11b markers more than PP2 alone, we speculate that PP2 may initiate a cascade of events (in place of RA), but RA may help drive these signaling events after initiation is achieved.

Fgr expression can be detected in R38+ and R38− only with combined PP2+RA treatment (Figure 5C). Compared to RA-treated WT HL60, this Fgr expression is of similar level in R38+ but lower in R38− (Figure 5C), indicating that increased Fgr expression may be correlated with CD38 expression. Fgr expression is also correlated with higher CD11b and p47^phox^ expression. The lack of induced Fgr with PP2 treatment alone again points to Lyn as the predominant Src-family kinase, as Lyn expression is better correlated with the induced signaling and differentiation-associated changes (c-Raf expression/phosphorylation, expression of Vav1, c-Cbl, Slp76, and G1/G0 cell cycle arrest) in these cells.

Much work remains to be done to understand how signaling is altered in these RA-resistant HL60 cells. In general, elucidation of c-Raf phosphorylation sites, MAPK feedback mechanisms and the role of specific Src-family kinases with non-overlapping functions is still needed. By initiating these fundamental inquiries first in model *in vitro* systems such as HL60, we can expedite and clarify our understanding of multiple, interconnected signaling pathways before progressing to *in vivo* systems.

## Supporting Information

Figure S1 **48 h and 72 h data for NBT Reduction after PP2 treatment.** To assess ROS production, control and PP2−, and/or RA-treated WT, R38+ and R38− HL60 cells were stimulated with TPA and analyzed by NBT reduction. Error bars represent standard error of at least three repeats. P values were calculated using a student’s t test and are compared between untreated respective control unless otherwise indicated. A: RA-resistant lines R38+ and R38− displayed no significant inducible ROS production during PP2, RA, or PP2+RA treatment at 48 h or 72 h. B: In RA-treated WT HL60, PP2 treatment decreased NBT reduction compared to RA alone (p<0.01 compared to control).
(TIF)
